# Supplementary material for: Winter occurrence and spawning characteristics of Pacific herring (Clupea pallasii) in Jinhae Bay: An integrated survey using acoustic monitoring, gillnet sampling, and environmental DNA
Source: PLoS One. 2026 Feb 4;21(2):e0338479. doi: 10.1371/journal.pone.0338479 (PMC12872025; doi:10.1371/journal.pone.0338479)
Supplement: Attachment — Submitted filename: Reviewers comments_Manuscript Number PONE-D-25-36896.docx [file pone.0338479.s001.docx]

**General comments**

The authors may benefit from language editing to enhance the clarity of their work and reduce ambiguity. A lot of the writing uses active voice, especially in the materials and methods, results and discussion sections of the work. The combination of acoustic telemetry, gill net surveys and eDNA is indeed interesting since these complementary methods allow for the triangulation of spawning events with greater precision than just using one approach. As such, it brings out the spatial and temporal dynamics of C. pallasii spawning. The study further lacks details in the methodology section thus impacting on reproducibility of the work.

The results for this study lacks statistical analyses to tease out any temporal differences in spawning activity and whether correlations exist between the different methods. Authors should consider adding appropriate statistical analyses to investigate differences, if any. For the discussion, the authors may consider revising and improving the discussion for clarity and flow from one paragraph to another. Finally, it would be great if the authors could demonstrate how study improves our knowledge on spawning ecology and how it could possibly be used to aid fisheries monitoring.

**Specific comments**

**Introduction**

Line 60 – 61: The following sentence is unclear:

“To protect C. pallasii resources, which are commercially and biologically important, since 2022, South Korea has implemented a minimum landing size of 20 cm [10].”

The authors may consider rephrasing to enhance clarity. “Resources” here is a bit vague.

Line 67-69: Similarly, the following sentence is also unclear:

“On the other hand, for accurate species identification, other survey methods need to be used in parallel, such as a gillnet catch survey or eDNA analysis.”

Also consider revising for clarity.

Line 79-86: While the aim is “…ascertaining fluctuations in C. pallasii occurrence and comprehensively analysing spawning season characteristics” and the objective is “…to ascertain the time and relatively volume of C. pallasii migration to Jinhae Bay for spawning,” the primary objective is not coming out clearly. The authors may consider rephrasing to highlight what the primary objective is since it is not clear whether the study is focused on only the spawning season(s) or if migration patterns are important as part of the spawning season.

**Materials and Methods**

Authors should consider using passive voice in the materials and methods section instead of active voice. All through the text, there was a mix of active and passive voice. For example, they should avoid using or starting with “To monitor…” (line 90), “To install…” (line 100) and re-write the sentences in passive voice.

Overall, this section needs revision to improve clarity.

Lines 90 – 94:

Firstly, the study lacks description. Authors to consider including a brief description of the study site before providing the sampling schedule.

Secondly, authors may consider having figure 1 appearing before table 1 under the Study Site and Schedule subheading, prior to WBAT installation and analysis of acoustic data subheading. Then Figure 1 could be referred to from within the proceeding subheading.

Authors should state how many stations for the study. Table 1 caption/title should be re-written for clarity.

Lines 99 – 125: Authors should consider re-writing in passive voice for clarity. While depth of the many sites have been mentioned in Table 1, it may be good for the authors to mention it in the WBAT installation process and for WBAT, Provide the full names of what it actually is then continue using abreviations. Furthermore, what parameters do the WBAT record? Please provide this in the text.

What was the mooring period for the installed WBAT, i.e., for how long were the WBAT installed for? This is missing.

Lines 127 – 135: Authors should consider re-writing this section in passive voice for clarity. While gear specifications have been mentioned, there lacks clarity.

For instance, what was the survey period, season, sampling frequency (e.g., daily, weekly, monthly, etc), environment (e.g., habitat type, etc)?

How were the gears deployed and are they standardized?

What was the total number of fish collected from the gill net survey? i.e., How was the catch handled (e.g., total number of fish caught/sampled, species identification as per reference guides, length, weight, sex and maturity stage)?

Lines 138 - 155: Authors should consider re-writing this section in passive voice for clarity. Authors should avoid using the active voice “To analyse C. pallasii eDNA …”and revise to passive voice. This appears in several instances in the sub-section. The sampling design for water sampling collection for eDNA is not clear.

Authors should clarify surface layer depth (e.g. is it 0 – 1 m?), bottom depth whether for each station water is collected (e.g. is it 8 m, 10 m, or 12 m, etc) or a single bottom depth if it was uniform across all stations.

For eDNA extraction, while QIAGEN DNeasy Blood and Tissue Kits were used, what was the protocol for extraction? Were manufacturer’s instructions followed or modified? Were there any blanks or control used during DNA extraction? In addition, the reasons for DNA extraction to compare to eDNA samples is not clear.

For PCR amplification, there is no mention of the primers used/developed, and if developed, how they were developed. There is also no mention of the thermal cycling conditions. Consider including all these.

Are there any statistical analysis conducted to identify peak spawning periods apart from the time series analysis（e.g. comparing seasonal/monthly differences in catch rates or eDNA signals）and was consistency between the different methods validate (e.g., is there any correlation between eDNA and WBAT acoustic, etc)? Authors should consider adding appropriate statistical analyses to investigate differences, if any.

**Results**

Authors should consider revising using passive voice for clarity and go directly to focussing on just the results. Also, considering that statistical analyses was not mentioned in the methods, are there any temporal differences in catch and size, differences in species and maturity stage proportions or eDNA signals that reflect spawning activity?

Lines 159 – 160: While water temperature has been mentioned as part of the results, authors have not mentioned how they arrived to this in the methods section.

Lines 211: Authors mentioned the presence of contamination in their eDNA results yet in line 157 of methods, they did mention on how analysis was conducted to prevent contamination. Why would there be contamination in the NC sample from mid-January but not in other NC samples? What was the role of the DNA extraction from tissue samples of the C. pallasii?

**Discussion**

While the discussion has compared the study results for the different methods used to assess Winter occurrence and spawning characteristics of Pacific herring with other studies, the paragraphs appear seem disjointed from one to the next. For instance, in 2nd paragraph, the authors discuss the 7 gillnet surveys but then discuss the again in the 6th paragraph right after discussion of the qPCR eDNA analysis results. Same instance can be said on WBAT discussion (4th and 7th paragraphs).

The authors may consider using the last paragraph of the discussion as the introducing paragraph of the Discussion since it provides a quick snapshot of the results before the results are discussed at length in the proceeding paragraphs.

**Conclusion**

The authors should consider writing the conclusion in a single paragraph with just the key findings, emphasising on how the benefits of using the complementary methods approach and any future applications or research where it can be used. Were there any limitations in the study? What are the future directions? How does this study or can this study provide more knowledge on spawning ecology and fisheries monitoring?
